# Modified osteotomy for treatment of forearm deformities (Masada IIb) in hereditary multiple osteochondromas: a retrospective review

**DOI:** 10.1186/s12891-021-04829-x

**Published:** 2021-11-10

**Authors:** Ge Yan, Guoxin Nan

**Affiliations:** 1 Department of Orthopaedics Children’s Hospital of Chongqing Medical University, China, Yuzhong District Zhongshan 2road 136#, 400014 Chongqing, China; 2 National Clinical Research Center for Child Health and Disorders, Ministry of Education Key Laboratory of Child Development and Disorders, Chongqing, 400014 China; 3 China International Science and Technology Cooperation base of Child development and Critical Disorders; Chongqing Key Laboratory of Pediatrics, Chongqing, 400014 China

**Keywords:** Hereditary multiple osteochondromas, Forearm deformity, Ulnar lengthening

## Abstract

**Purpose:**

Approximately 30% of patients with hereditary multiple osteochondromas (HMO) have forearm deformity and dysfunction. The aim of this retrospective study was to review our experience with the surgical treatment of children with HMO and Masada IIb forearm deformities.

**Methods:**

Data of eight children treated for HMO Masada IIb forearm deformity at our hospital between 2015 and 2019 were collected from the hospital records and retrospectively reviewed. All patients underwent ulnar lengthening by distraction osteogenesis using either the Orthofix or Ilizarov external fixator. Range of movements at the elbow and wrist joints, and forearm supination/pronation, before and after the operation were recorded. Radiographs were evaluated by the Fogel method, and wrist joint function by the Krimmer method.

**Results:**

Follow-up radiographs showed significant improvement in relative ulnar shortening after treatment (pre-operative 9.23 ± 5.21 mm; post-operative 0.33 ± 4.13 mm). Changes in radial articular angle (pre-operative 33.55° ± 3.88° to 32.78° ± 6.57°) and carpal slip (pre-operative 45.00% ± 19.09%; post-operative 43.13% ± 16.68%) were not significant. Elbow flexion and extension, wrist flexion and extension, ulnar and radial deviation at wrist, and forearm rotation were significantly improved after surgery. Wrist function was graded as excellent in seven patients and as good in one patient. One patient treated with the Ilizarov external fixator had poor radial head reduction.

**Conclusion:**

Ulnar lengthening with distraction osteogenesis is an effective treatment for HMO Masada IIb deformities. The optimum site for ulnar osteotomy appears to be at the proximal one-third to one-fourth of the ulna.

**Supplementary Information:**

The online version contains supplementary material available at 10.1186/s12891-021-04829-x.

## Introduction

**Fig. 1:**
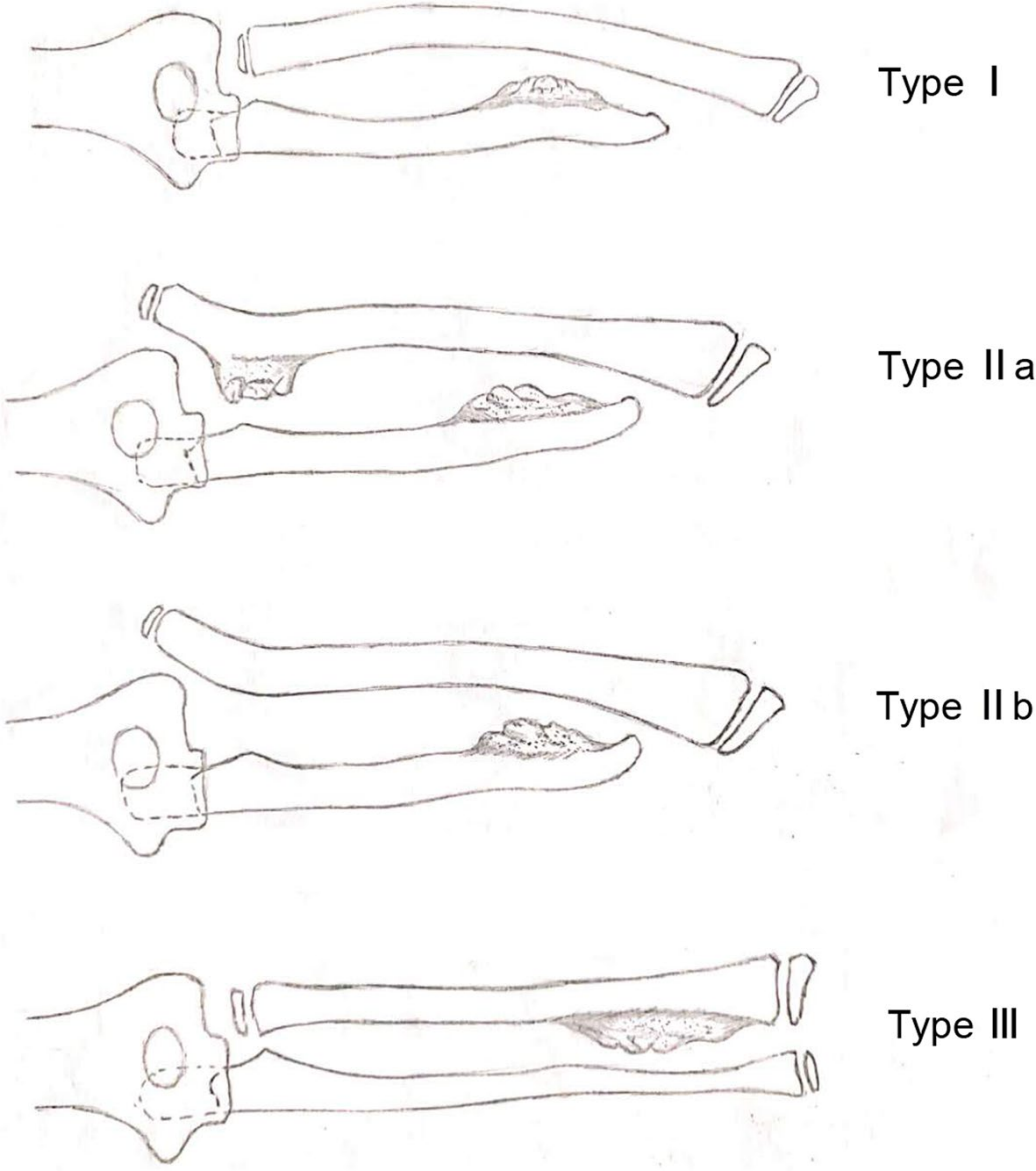
Masada classification

Hereditary multiple osteochondroma (HMO) is an autosomal dominant benign tumour that affects cartilage and bone [1, 2]. It results from mutations of *EXT1* and *EXT2* [3], with the former causing more severe disease [1]. The tumor, usually multiple, arises from the metaphysis or diaphysis of the long bones of the extremities and is covered by a cartilage cap. Because the tumour interferes with normal bone growth, skeletal deformities are common, and most patients have abnormal limb lines and dysfunction. About 30% of patients with HMO will have forearm deformities [4, 5] that directly affect movements at the elbow joint and wrist joint and forearm supination/pronation. The deformities include ulnar bending, ulnar shortening, elbow varus, and wrist joint deformities [2, 4, 6, 7]. Masada et al. [8] classified forearm deformities caused by HMO into three types based on the presence or absence of radial head dislocation and the site of the osteochondroma; type II was divided into two subtypes (Fig. 1).

Surgery is usually required for type II deformity. Lengthening of the ulna by distraction osteogenesis—using the Orthofix external fixator or the Ilizarov annular external fixator—can help correct the radial head dislocation and improve the function of forearm rotation. While treatment outcomes are generally good, in some cases, the radial head may not be completely reduced or the wrist joint deformity may even be aggravated after ulnar lengthening. We believe that the choice of the osteotomy site is key to the success of the operation.

The aim of this study was to retrospectively review our experience with the treatment of children with HMO Masada IIb forearm deformity.

## Materials and methods

### Patients

The study sample comprised patients with HMO and Masada IIb forearm deformity who were treated with ulnar lengthening by distraction osteogenesis at our hospital between January 2015 and June 2019. Patients followed up for less than 10 months, and those with incomplete data were excluded. The pre-operative and follow-up data of these patients were retrieved from the hospital records and retrospectively reviewed.

The ethics committee of our hospital approved the study. Written informed consent was obtained from the patients’ parents or guardians before surgery.

The Orthofix external fixator was used for four patients (two males and two females; mean age, 10.75 years). These patients were followed up for a mean duration of 22.25 months after the surgery; the mean duration of ulna distraction was 6.0 months. The Ilizarov external fixator was used for four patients (two males and two females; mean age, 8.25 years). These patients were followed up for a mean duration of 22.25 months after surgery; the mean duration of ulna distraction was 5.75 months. Table 1 summarizes the characteristics of the patients.

**Table 1:** Characteristics of the patients

Patient	Age (years)	Sex	Side	Method	Duration of ulnar distraction (months)	Follow-up period (months)
1	11	M	Left	Orthofix	7	37
2	7	F	Right	Ilizarov	3	29
3	13	M	Right	Orthofix	8	12
4	7	M	Right	Ilizarov	9	15
5	9	F	Right	Ilizarov	6	35
6	10	M	Right	Ilizarov	5	10
7	9	F	Right	Orthofix	6	30
8	10	F	Left	Orthofix	3	10

### Evaluation methods

#### Radiographic evaluation

**Fig. 2:**
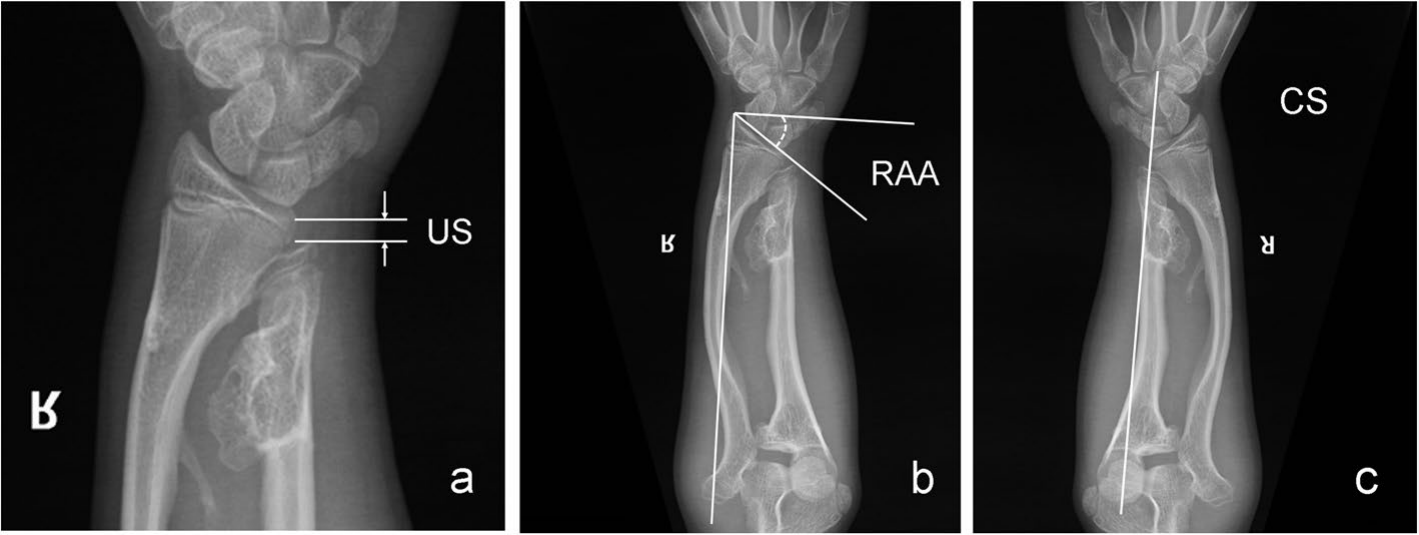
Radiographic evaluation indices proposed by Fogel et al.: **a** Relative ulnar shortening. **b** Radial articular angle. **c** Ulnarward carpal slip. **a** Relative ulnar shortening (RUS) is measured with the perpendicular line drawn from the distal end of the ulna to the linear axis of the forearm. **b** The radial articular angle (RAA) is the angle between two constructed lines: one along the articular surface of the radius and the other perpendicular to a line that bisects the head of the radius and passes through the radial edge of the distal radial epiphysis. The normal RAA is 15°-30°. **c** Ulnarward carpal slip (CS) or displacement of the lunate off the radius is measured as the percentage of contact of the lunate with the radius. An axial line drawn from the center of the olecranon through the ulnar edge of the distal radius normally bisects the lunate. Carpal slip is considered abnormal when the lunate is displaced ulnarward by > 50%

Fogel et al. [9] proposed three indices for evaluation of severity of forearm deformity in HMO: relative ulnar shortening (RUS), radial articular angle (RAA), and carpal slip (CS) (Fig. 2) [10, 11]. RUS helps to evaluate the success of ulna lengthening after surgery, while RAA and CS help evaluate the correction of radius curvature and ulnar deviation of the wrist joint, respectively, after ulnar lengthening.

The criteria of Sachar and Mih were used to classify radial head position before and after surgery [12]. According to these criteria, the radial head is in the normal position if a line coincident with the longitudinal axis of the proximal aspect of the radius passes through the center of the capitellum on both anteroposterior and lateral radiographs. If this line passes through the capitellum but not through its center, the radial head is classified as subluxated. If this line does not pass through the capitellum, the radial head is classified as dislocated.

#### Functional evaluation

Flexion and extension at the elbow, flexion and extension of the wrist, ulnar and radial deviation at the wrist, and pronation/supination of the forearm were recorded before surgery and during follow-up. Wrist function was rated according to the four criteria proposed by Krimmer et al. [13]: percentage of grip power, range of movement of the wrist, pain, and activity of the hand (Table 2).

**Table 2:** Krimmer criteria for assessment of wrist function

Percentage of grip power (%)			Score
0–25			0
> 25–50			10
> 50–75			20
> 75–100			30
Range of wrist motion (°)			
Extension/Flexion	Ulnar/Radial deviation	Supination/Pronation	Score
≤ 30	≤10	≤80	0
> 30–60	> 10–35	> 80–110	10
> 60–100	> 35–50	> 110–140	15
> 100	> 50	> 140	20
Pain			Score
Severe			0
Moderate pain at rest			10
Mild pain during activity			15
No pain			20
Restriction of activities			Score
Severe			0
Moderate			10
Mild			20
None			30
Overall outcome			Total score
Excellent			> 80–100
Good			> 65–80
Fair			> 50–65
Poor			0–50

### Surgical technique

All procedures were performed under general anesthesia and tourniquet control. For patient who received Orthofix, a longitudinal incision was made on the forearm and osteotomy was performed at the proximal one-third to one-fourth of the ulna. The proximal and distal ends of the ulnar osteotomy were fixed with two screws to the Orthofix external fixator. For patients who received Ilizarov, a longitudinal incision made on the lateral forearm. Due to the large volume of the ring frame, osteotomy was sometimes performed at the proximal one-half to one-third of the ulna. The proximal and distal ends of the ulnar osteotomy were fixed to the Ilizarov rings with two Kirschner wires each.

In both groups, distraction treatment was started on day 3 after the operation, with 0.25 mm of lengthening every 6 h. Radiographs were reviewed regularly to assess the lengthening and bone growth. The ulna lengthening program was adjusted according to the individual situation.

### Statistical analysis

SPSS 22.0 (IBM Corp., Armonk, NY, USA) was used for the statistical analyses. All the variables were assessed by Kolmogorov-Smirnov test for normality. Changes in RUS, RAA, range of flexion and extension at the elbow, forearm supination, wrist flexion, and wrist ulnar deviation after surgery were assessed by the paired *t*-test. Changes in CS, forearm pronation, wrist extension, and wrist radial deviation were assessed by the Wilcoxon test. *P* < 0.05 was considered statistically significant.

## Results

### Follow-up

All resected tumours were confirmed to be benign osteochondromas on pathological examination. Mean follow-up was for 22.25 months (range, 10–37 months). The mean period of ulnar distraction was 5.88 months (3–9 months).

### Radiographic outcome

Tables 3 and 4 show the changes in RUS, RAA, and CS. The mean RUS was significantly improved after surgery (*P* < 0.001); however, the changes in RAA and CS were not statistically significant (*P* = 0.64 and *P* = 0.83, respectively).

**Table 3:** Radiographic indices before surgery and at last follow-up

Patient	RUS (mm)		RAA (°)		CS (%)	
	Before surgery	At last follow-up	Before surgery	At last follow-up	Before surgery	At last follow-up
1	6.9	1.6	35.8	36.9	65	40
2	18.0	4.8	35.8	44.0	5	30
3	2.5	−6.6	37.1	32.2	50	10
4	13.8	2.3	28.0	28.5	50	50
5	10.3	−2.5	37.5	34.1	60	50
6	3.0	−3.5	27.6	21.1	50	65
7	10.4	1.5	32.7	31.7	30	50
8	8.9	5.0	33.9	33.7	50	50

*RUS* Relative ulnar shortening, *RAA* Radial articular angle, *CS* Carpal slip

**Table 4:** Changes in radiographic parameters after treatment

Parameter	Before surgery	At last follow-up	*P*
RUS (mm)	9.23 ± 5.21	0.33 ± 4.13	< 0.001
RAA (°)	33.55 ± 3.88	32.78 ± 6.57	0.64
CS (%)	45.00 ± 19.09	43.13 ± 16.68	0.83

*RUS* Relative ulnar shortening, *RAA* Radial articular angle, *CS* Carpal slip

Table 5 shows the radial head position before and after the surgery in each patient. While reduction was finally achieved in three patients, the dislocations/subluxations improved in the other five patients.

**Table 5:** Radial head position before and after the surgery

Patient	Before surgery	At last follow-up
1	Dislocated	Reduced
2	Subluxated	Reduced
3	Dislocated	Subluxated
4	Dislocated	Subluxated
5	Subluxated	Reduced
6	Subluxated	Subluxated
7	Dislocated	Subluxated
8	Dislocated	Subluxated

### Functional outcomes

Elbow flexion and extension, wrist flexion and extension, ulnar and radial deviation at the wrist, and forearm supination/pronation improved significantly in patients treated with both types of external fixators (*P* < 0.05 for all; Tables 6, 7). According to the Krimmer et al. criteria, the improvement was excellent in seven patients and good in one patient; no patient was graded as fair or poor.

**Table 6:** Range of motion before surgery and at last follow-up for each patient

Patient	Elbow joint		Forearm				Wrist joint							
	Flexion and extension (°)		Pronation (°)		Supination (°)		Flexion (°)		Extension (°)		Ulnar deviation (°)		Radial deviation (°)	
	Before surgery	At last follow-up	Before surgery	At last follow-up	Before surgery	At last follow-up	Before surgery	At last follow-up	Before surgery	At last follow-up	Before surgery	At last follow-up	Before surgery	At last follow-up
1	120	135	55	65	65	70	45	55	45	50	30	20	15	25
2	130	135	50	55	50	60	40	50	45	50	45	25	15	30
3	110	115	35	55	40	55	40	65	30	40	30	20	25	35
4	90	110	40	55	50	65	30	45	35	45	40	30	15	25
5	130	130	40	60	60	75	30	55	35	50	50	35	15	30
6	115	125	55	70	55	70	45	60	55	65	50	45	20	30
7	95	115	40	60	60	70	35	60	45	60	35	25	15	25
8	100	105	40	50	55	60	45	50	45	50	40	35	15	20

**Table 7:** Mean range of motion before surgery and at last follow-up

Measurement		Before surgery	At last follow-up	*P*
Elbow joint	Flexion and extension (°)	111.25 ± 15.29	121.25 ± 11.57	0.01
Forearm	Pronation (°)	44.38 ± 7.76	58.75 ± 6.41	0.01
	Supination (°)	54.38 ± 7.76	65.63 ± 6.78	< 0.001
Wrist joint	Flexion (°)	38.75 ± 6.41	55.00 ± 6.55	< 0.001
	Extension (°)	41.88 ± 7.99	51.25 ± 7.91	0.01
	Ulnar deviation (°)	40.00 ± 8.02	29.38 ± 8.63	< 0.001
	Radial deviation (°)	16.88 ± 3.72	27.50 ± 4.63	0.01

### Complications

**Fig. 3:**
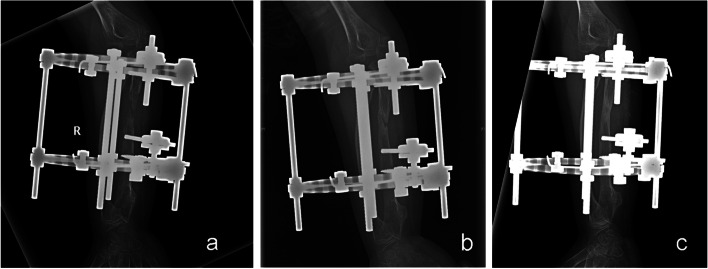
Radiographs of patient 5: **a** There is delayed healing, with non-union at the osteotomy site at the end of 2 months of postoperative distraction. **b** The non-union healed gradually with application of sustained reverse pressure over 2 months. **c** The distraction was re-applied for another 2 months

At the final follow-up, two patients (Patients 4 and 8) had persistent mild pain. Two patients (Patients 3 and 4) had pin-track infection; in both cases the infection was controlled by pin care and oral antibiotics. One patient (Patient 5) had delayed healing at the osteotomy after 2 months of postoperative distraction (Fig. 3a). The non-union healed gradually with application of sustained reverse pressure over 2 months (Fig. 3b), following which distraction was re-applied for another 2 months (Fig. 3c). No neurovascular complication occurred in any patients.

## Discussion

In Masada IIb HMO, shortening of the ulna and dislocation of the radial head are mostly responsible for the forearm deformity and dysfunction. Simple resection of the osteochondroma is usually not sufficient as the deformity tends to recur. Previous research has shown that ulnar lengthening can prevent the occurrence of radial head dislocation [14]. Matsubara et al. [15] were the first to show that if patients in the growing stage have to undergo surgery due to poor forearm function, satisfactory outcomes can be obtained by excessive lengthening of the ulna; this appears to be sufficient for improving the range of motion at the elbow and wrist joints. For patients with wrist deformity after ulnar lengthening, distal radius osteotomy combined with Epibloc™-system fixation is a good treatment option [16].

**Fig. 4:**
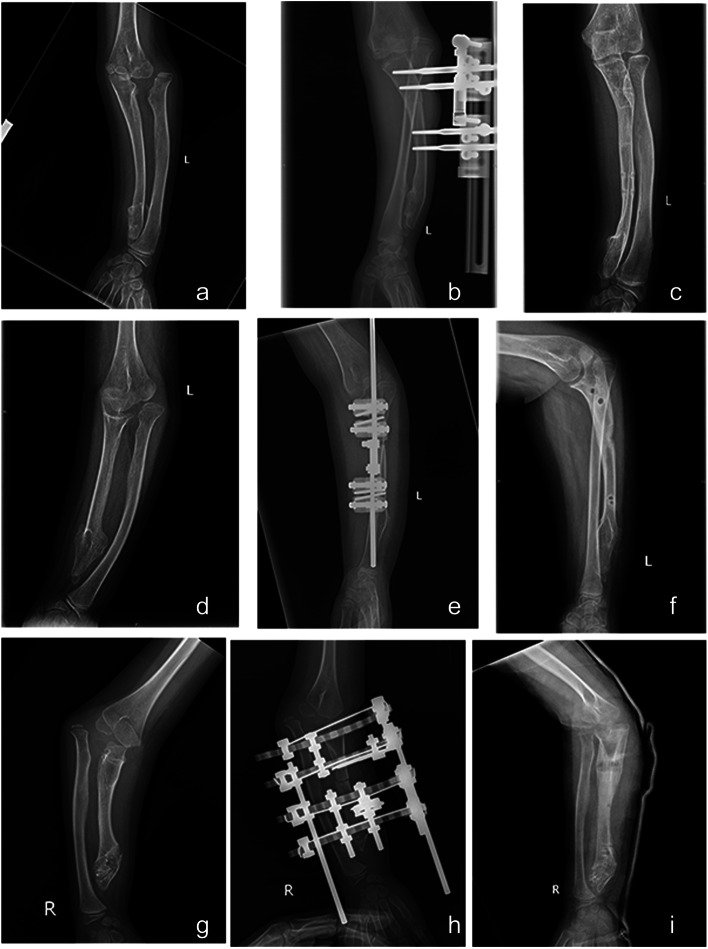
**a**-**c** Pre-operative and post-operative radiographs of Patient 1. **d**-**f** Pre-operative and post-operative radiographs of Patient 8. **g**-**i** Pre-operative and post-operative radiographs of Patient 4

Recent studies have confirmed that ulnar lengthening combined with osteochondroma resection is effective treatment for type I Masada forearm deformity with relative ulnar shortening [11, 17]. The efficacy of ulnar lengthening for correcting radial head dislocation in type IIb Masada malformation has not yet been established. In fact, good reduction of the radial head is difficult to achieve during treatment of type IIb Masada deformity. In the present series, there was significant improvement in range of movements overall; however, reduction of the radial head was not successful in all cases. One patient (Patient 1, Fig. 4a) with obvious dislocation of the radial head, underwent ulnar distraction with an Orthofix external fixator (Fig. 4b) for 7 months. After the Orthofix was removed (Fig. 4c), the patient was followed up for 9 months, over which period the appearance improved significantly. Another patient (Patient 8, Fig. 4d) with obvious dislocation of the radial head, received Orthofix distraction lengthening (Fig. 4e). The Orthofix was removed after 3 months (Fig. 4f). Over the next 3 months, although the appearance improved slightly, range of motion at the wrist and elbow joints did not improve significantly. A third patient (Patient 4, Fig. 4g), also with obvious dislocation of the radius head, received the Ilizarov external fixator (Fig. 4h). After regular lengthening for 9 months, although ulnar lengthening was satisfactory, the radial head was poorly repositioned (Fig. 4i). After removal of the fixator, the child was followed up for 3 months, over which period the range of motion at the wrist and elbow joints improved only slightly.

**Fig. 5:**
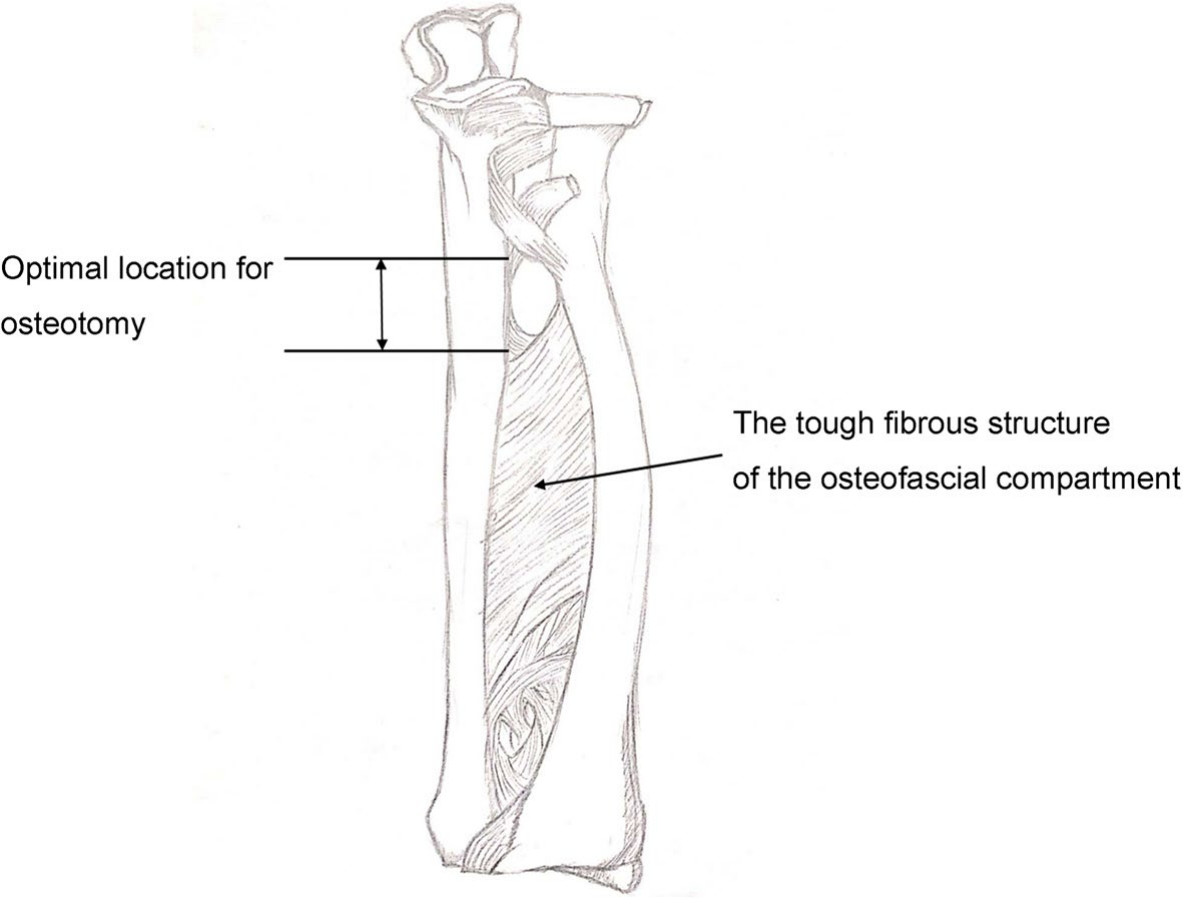
Anatomical structure of the osteofascial compartment

In the current study, radial head reduction could not be achieved in some children despite successful lengthening of the ulna with external fixator distraction. We believe that successful radial head reduction depends on the location of the ulnar osteotomy. The osteofascial compartment (Fig. 5) in the forearm contains tough inelastic fibrous tissue that connects the ulna and radius. If the ulna osteotomy site is relatively distal, the radius will move distally during ulnar distraction because the traction effect will be transmitted to the radius via the fibrous structures in the osteofascial compartment. This will result in failure of reduction of the radial head; moreover, the relative shortening of the distal ulna will not be corrected. The optimal position for the osteotomy appears to be between the proximal one-third and one-fourth of the ulna, where the fibrous connection between the ulna and radius is relatively weak. In our series, we found that a relatively distal osteotomy was more likely when the Ilizarov external fixator was used.

Osteotomy at the proximal ulna has another important advantage. The proportion of cancellous bone is high in this region. Cancellous bone is more osteogenic than cortical bone because of the presence of spaces within its structure, which allows the diffusion of nutrients and limited revascularization by microanastomosis of its circulating vessels [18, 19]. Thus, bone healing after osteotomy is better, and nonunion less likely, when osteotomy is performed at the proximal ulna. If forearm non-union occurs, intramedullary nailing, possibly combined with tricortical autologous bone grafting, is an effective treatment option [20].

The evaluation indices proposed by Fogel et al. [9] (i.e., RUS, RAA, and CS) are widely used in research on forearm malformation in HMO [8, 10, 11, 21]. In the present study we found that ulnar lengthening generally resulted in marked improvement of RUS. RAA did not change much, while the changes in CS were inconsistent—with marked change in some patients and no change at all in others. Thus, our study suggests that, in patients with Masada type IIb deformity, ulnar lengthening can be achieved, but the bone deformity is difficult to improve. Further, RAA and CS do not appear to be of use for evaluating therapeutic effect in these deformities. Accurate evaluation will require consideration of RUS and radial head reduction along with degree of improvement in range of motion at the elbow and wrist.

Our research is limited by its retrospective nature, small sample size, and short follow-up time. The study findings must be confirmed in long-term prospective randomized controlled studies.

## Conclusion

In Masada IIb HMO, ulna osteotomy and distraction lengthening appears to be effective for reducing the dislocated radial head, correcting deformity, and improving elbow and wrist mobility. The best therapeutic effect is achieved with an osteotomy between the proximal one-third and one-fourth of the ulna.

## Supplementary Information

**Additional file 1.** STROBE Statement.

## Data Availability

The datasets used and/or analysed during the current study are available from the corresponding author on reasonable request.

## Abbreviations

HMO: Hereditary multiple osteochondromas
RUS: Relative ulnar shortening
RAA: Radial articular angle
CS: Carpal slip
